# Haplotype-based allele mining in the Japan-MAGIC rice population

**DOI:** 10.1038/s41598-018-22657-3

**Published:** 2018-03-12

**Authors:** Daisuke Ogawa, Eiji Yamamoto, Toshikazu Ohtani, Noriko Kanno, Hiroshi Tsunematsu, Yasunori Nonoue, Masahiro Yano, Toshio Yamamoto, Jun-ichi Yonemaru

**Affiliations:** 1 Institute of Crop Science, National Agricultural and Food Research Organization (NARO), Tsukuba, Japan; 2 Agrogenomics Research Centre, National Institute of Agrobiological Sciences (NIAS), Tsukuba, Japan

## Abstract

Multi-parent advanced generation inter-cross (MAGIC) lines have broader genetic variation than bi-parental recombinant inbred lines. Genome-wide association study (GWAS) using high number of DNA polymorphisms such as single-nucleotide polymorphisms (SNPs) is a popular tool for allele mining in MAGIC populations, in which the associations of phenotypes with SNPs are investigated; however, the effects of haplotypes from multiple founders on phenotypes are not considered. Here, we describe an improved method of allele mining using the newly developed Japan-MAGIC (JAM) population, which is derived from eight high-yielding rice cultivars in Japan. To obtain information on the haplotypes in the JAM lines, we predicted the haplotype blocks in the whole chromosomes using 16,345 SNPs identified via genotyping-by-sequencing analysis. Using haplotype-based GWAS, we clearly detected the loci controlling the glutinous endosperm and culm length traits. Information on the alleles of the eight founders, which was based on the effects of mutations revealed by the analysis of next-generation sequencing data, was used to narrow down the candidate genes and reveal the associations between alleles and phenotypes. The haplotype-based allele mining (HAM) proposed in this study is a promising approach to the detection of allelic variation in genes controlling agronomic traits in MAGIC populations.

## Introduction

Phenotypes differ according to the different alleles of a gene and gene-gene interactions. In genetic studies, bi-parental populations, such as recombinant inbred lines (RILs) and backcrossed inbred lines, are useful because of their simple inheritance patterns. However, the richness of their allelic and phenotypic variations is limited. To overcome this limitation, the collaborative cross (CC) of a multi-parental inter-mated population descending from eight mouse strains has been proposed^1^; the resulting population has more diverse alleles than the bi-parental population and is a useful resource for understanding the complex biology of mammals. Multi-parental intermated populations, known as multi-parent advanced generation inter-cross (MAGIC) lines have also been developed in plants, such as *Arabidopsis thaliana*^2^, tomato^3^, faba^4^, wheat^5,6^, barley^7^, maize^8^ and rice^9–11^. Single-nucleotide polymorphism (SNP) array and genotyping-by-sequencing (GBS) analysis based on next-generation sequencing (NGS) can assist in characterizing the genomic constitution and genetic architecture of these lines^12,13^. Linkage disequilibrium (LD) is reduced in MAGIC populations compared with that in bi-parental populations^6,8,11^. Genetic studies on MAGIC populations have revealed quantitative trait loci (QTLs) that control the transition from the vegetative to reproductive stage^2,7,10,11^, yield^3,5,8,14^, grain quality^9^, morphological development^2,5,8,10,11,15,16^, and responses to abiotic and biotic stresses^4,9,17^.

Genome-wide association studies (GWAS) using high-density SNPs have been applied to MAGIC populations^3,9–11^. However, the causative genes of a phenotype have rarely been identified. Therefore, improvements in GWAS methods are needed. Deep resequencing analysis can detect nucleotide variations in all genes of founders and can reveal the putative functions of the alleles. This information should facilitate the identification of causative genes. In the traditional GWAS methodology, the associations of a phenotype with SNPs are investigated using statistical models, such as mixed linear models^18,19^. SNPs include founder-specific and founder-shared randomly aligned SNPs, and genetically linked SNPs on a chromosome are integrated as haplotypes. The haplotypes in a MAGIC population might be informative for GWAS because they represent recombined chromosomal segments derived from founders.

In this study, we developed and characterized the Japan-MAGIC (JAM) population, a new MAGIC population descended from eight high-yielding rice cultivars in Japan. Genome-wide SNPs obtained by GBS analysis in the JAM population were used to translate the SNP data into haplotype blocks. We also determined the full allelic diversity from high-depth sequencing data of the eight founders. Finally, we evaluated the proposed allele mining method using the haplotype data of the JAM lines and the allelic information of the eight founders.

## Results

### Production of the JAM and four RIL populations

**Figure 1:**
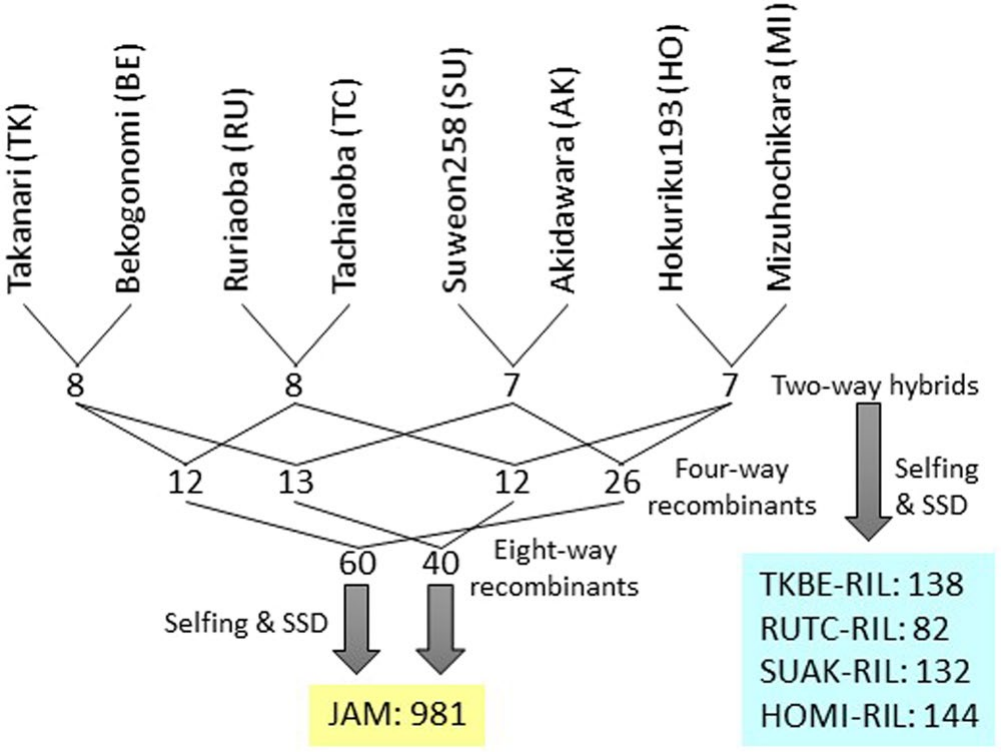
Development of the Japan-MAGIC (JAM) population and RILs. The JAM population was produced from eight cultivars through crossing, selfing and single-seed descent (SSD) steps. V-shaped lines indicate parents in each crossing. The numbers of two-way hybrids and four- and eight-way recombinants used to develop the JAM population are shown. Four types of recombinant inbred lines (RILs) were developed from two-way hybrids. The number of lines in the JAM population and the number of RILs of each type are indicated.

**Figure 2:**
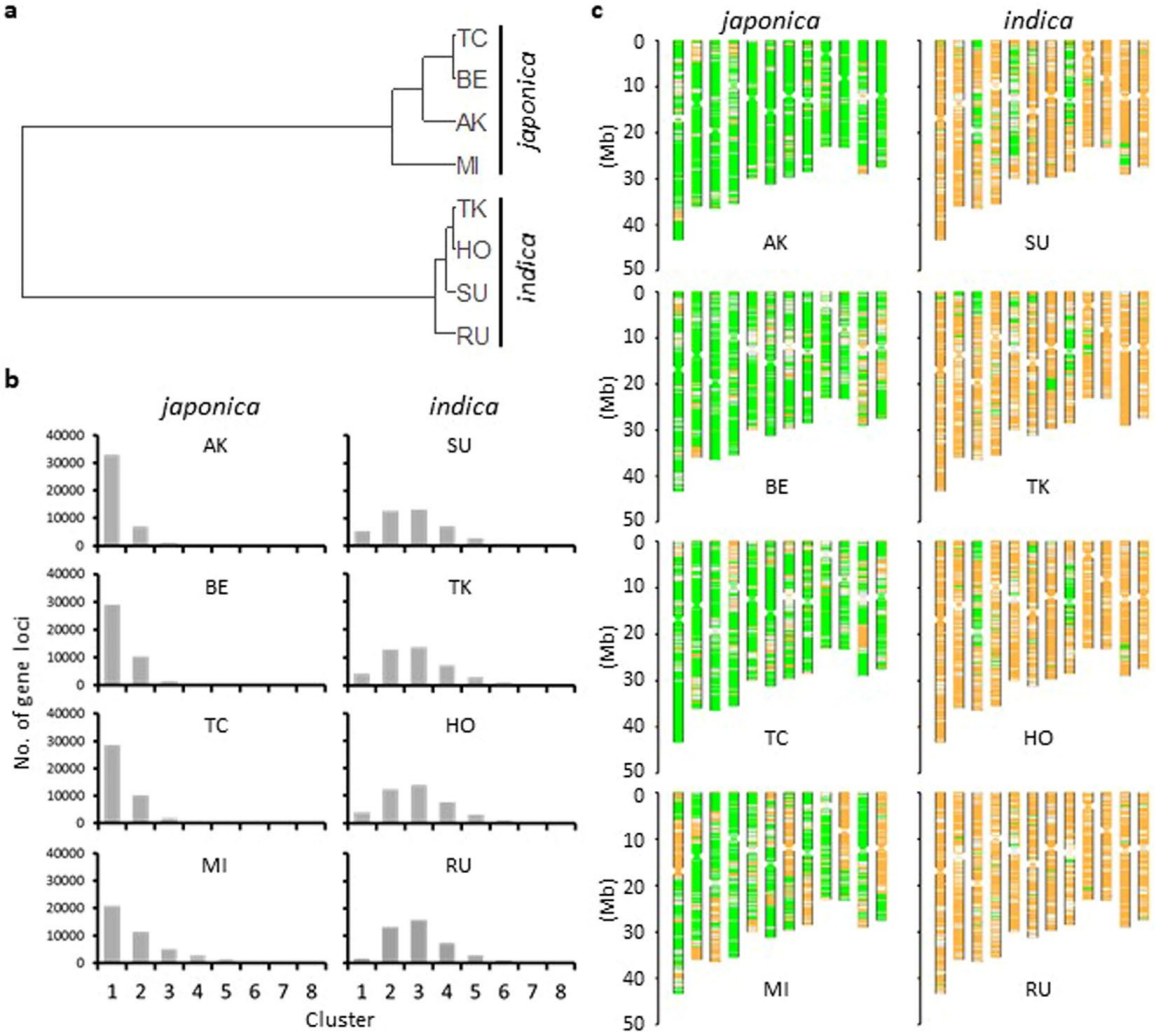
Categorization of annotated genes in the eight founders. (**a**) Hierarchical cluster classification of the founders according to Ward’s method. Categorization of alleles was performed using NGS data and considering the severity of allelic variations based on the SNP effect (SnpEff) category. (**b**) Number of loci in each gene cluster. (**c**) Position of the categorized alleles on chromosome maps. Cluster 1, Cluster 2 and Clusters 3–8 are indicated in green, white and orange, respectively.

We used eight cultivars as parents of the JAM lines: Ruriaoba (RU), Hokuriku 193 (HO), Takanari (TK), Suweon 258 (SU), Mizuhochikara (MI), Bekogonomi (BE), Tachiaoba (TC) and Akidawara (AK) (Fig. 1 and Supplementary Fig. 1). From the whole-genome sequencing data and variant analysis of all annotated genes, the former four cultivars were identified as *indica* and the latter four as *japonica* (Fig. 2a). We first crossed the *indica* and *japonica* cultivars to produce seeds of four types, called two-way hybrids (Fig. 1). These hybrids were crossed to produce four-way recombinants. Intermating of the four types of four-way recombinants produced 100 eight-way recombinants. We attempted to produce 1,000 JAM lines by developing 10 lines from each eight-way recombinant and then using the single-seed descent (SSD) method. Finally, we produced F_6_ seeds from 981 JAM lines. The four types of two-way hybrids were also used to produce four bi-parental RIL populations: TKBE (TK × BE), RUTC (RU × TC), SUAK (SU × AK) and HOMI (HO × MI) (Fig. 1).

### High phenotypic variation in the JAM population

**Figure 3:**
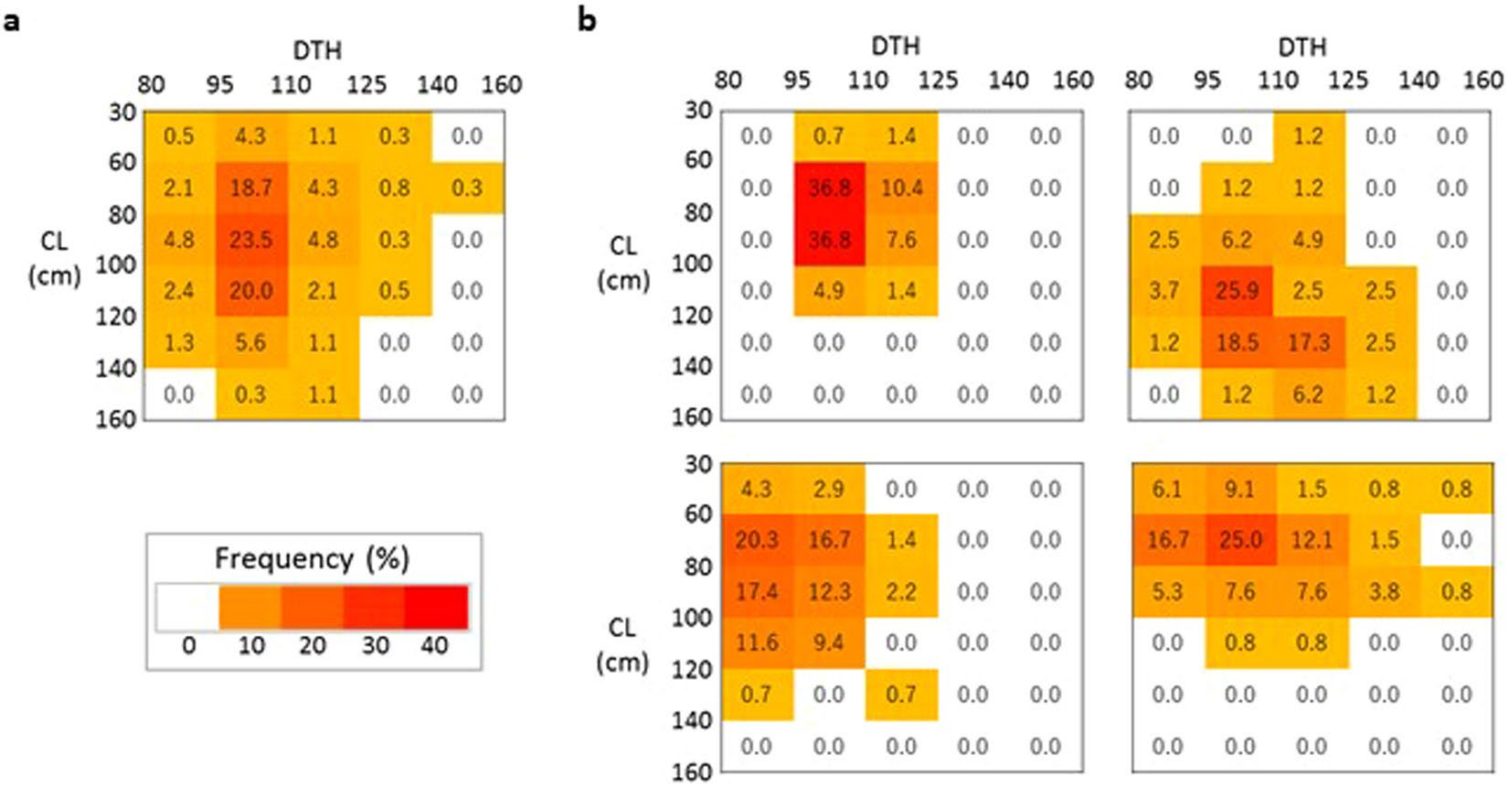
Phenotypic diversity of 376 JAM lines and RILs. (**a**) JAM lines. (**b**, top left) HOMI-RILs, (**b**, bottom left) TKBE-RILs, (**b**, top right) RUTC-RILs, and (**b**, bottom right) SUAK-RILs. Phenotypic diversity is shown as heat maps of the frequencies of lines with different combinations of DTH (days to heading) and CL (culm length). The DTH range was divided into five categories and that of CL into six categories. The frequency of lines with each DTH–CL combination is shown in the centre of each cell.

We assessed variation in two traits: days to heading (DTH) and culm length (CL). The values of both RILs and JAM lines (DTH: 90–142 days; CL: 41–158 cm) were distributed beyond the parental ranges, indicating transgressive segregation (Supplementary Fig. 2). Unlike the values of RILs, those of the JAM lines were distributed widely in the centre of the graph. To analyse the variation using a different approach, we classified the values into five DTH and six CL categories (30 classes in total) and calculated the frequency of JAM lines in each class (Fig. 3). The JAM lines occupied 22 classes, whereas RILs occupied fewer classes (8, HOMI-RILs; 12, TKBE-RILs; 17, RUTC-RILs; and 16, SUAK-RILs). The JAM distribution appears to be an overlay of those of the four RILs. These results indicate that the JAM population has higher phenotypic diversity than each of the RILs.

### Genome structure of the JAM population revealed by GBS analysis

**Figure 4:**
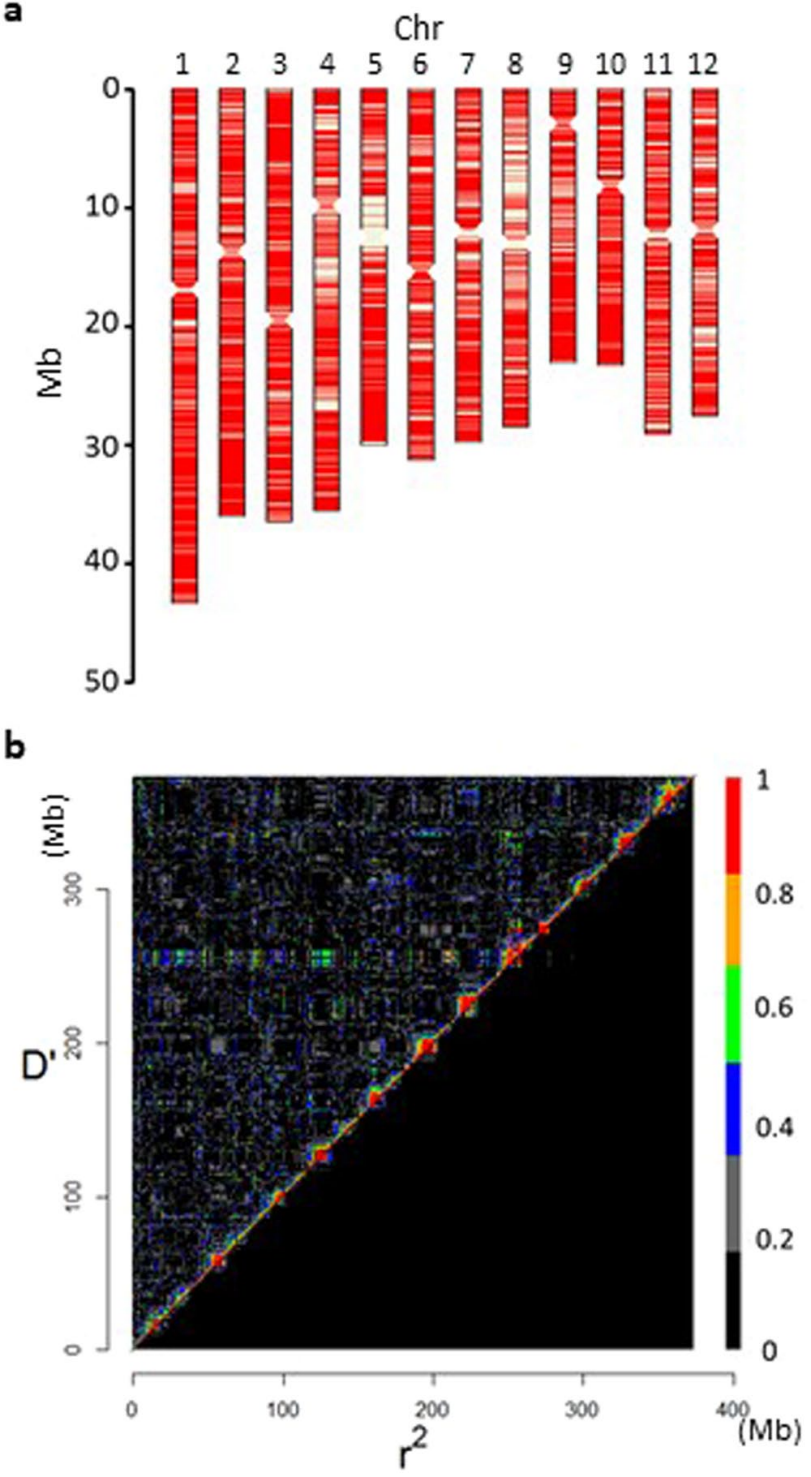
Genotypic characteristics of the 376 JAM lines. (**a**) Positions of 16,345 SNP alleles detected by genotyping-by-sequencing analysis. (**b**) Heatmap of linkage disequilibrium decay of 2,355 randomly selected SNP alleles (1/7 of all SNP alleles) according to the physical map over 12 chromosomes. Physical positions are indicated as cumulative positions (Mb) from chromosome 1 to 12. D’ and r^2^ are shown above and below the diagonals, respectively.

Using GBS, we analysed the eight founders and 376 F_5_ JAM lines, which were selected by taking the origins of the eight-way recombinants into account. We extracted SNPs according to the following criteria: minor allele frequency of more than 0.0625 (1/16), no third allele and less than 5% of missing values in all lines; the missing values were imputed with the BEAGLE program (http://faculty.washington.edu/browning/beagle/b3.html). We obtained the data for 16,345 SNP alleles distributed on all chromosomes with high density (Fig. 4a).

To analyse LD, we used 2,355 randomly selected SNPs. The mosaic pattern of high-LD pixels showed the regions of low recombination frequency in the centromeres and the surrounding sequences (Fig. 4b and Supplementary Fig. 3). Relatively high LD was detected in the centromeric regions of chromosomes 5 and 7–12 (Supplementary Fig. 3), although LD values calculated between the SNP pairs within 5-Mb windows were lower on chromosomes 5 and 8 (Supplementary Fig. 4). The lower SNP density in these regions resulted in apparently higher LD values.

### Proportions of haplotypes from the eight founders in the JAM lines

**Figure 5:**
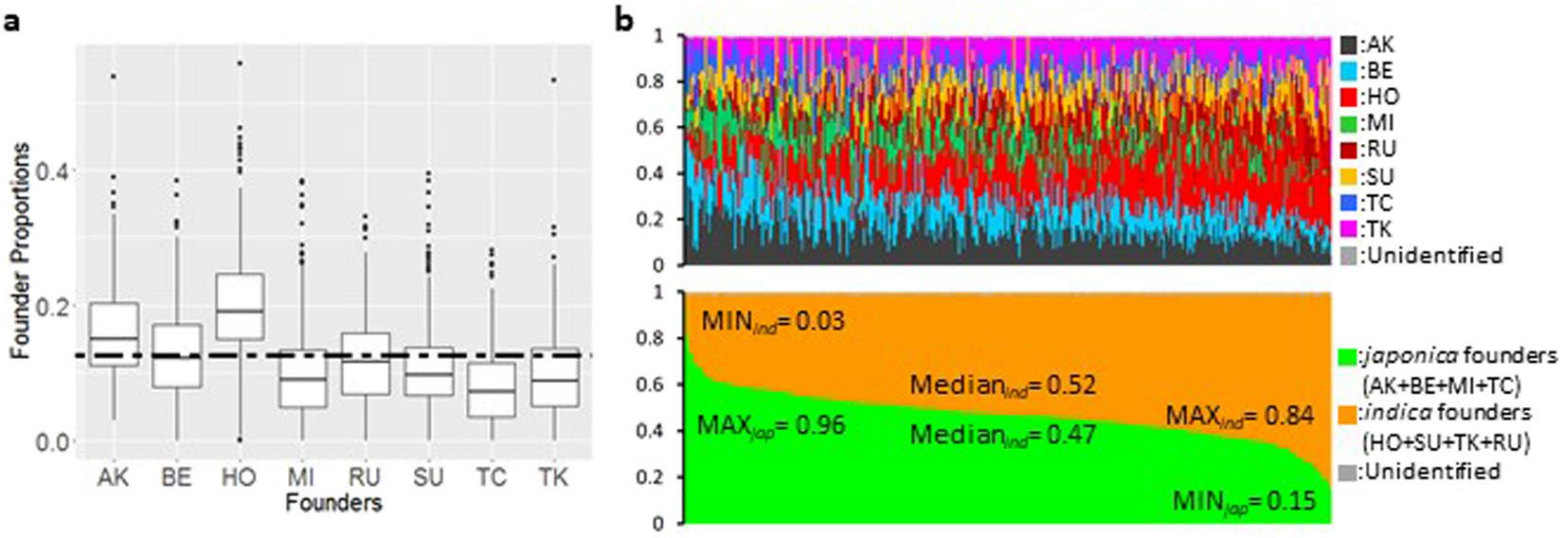
Population structure of the 376 JAM lines. (**a**) Box plots of the founder genomes in the 376 JAM lines. Abbreviations of the eight founders are as in Fig. 1. A chain line indicates 0.125. (**b**) The upper panel indicates the rate of the estimated eight haplotype fractions in each JAM line. The bottom panel indicates the rates of two fractions derived from the four *japonica* founders (AK + BE + MI + TC) and four *indica* founders (HO + SU + TK + RU). Each vertical bar in the two panels represents a single line in the JAM population and is arranged in order of the fraction values of *japonica/indica* founders.

Haplotypes in the JAM lines were estimated using the method proposed for haplotype prediction in an *Arabidopsis* MAGIC population (see the *Methods* section). In this work, the haplotypes at each SNP position are defined as types of genomes derived from the 8 founders. The proportion of haplotypes from the eight founders in the JAM population differed depending on genomic position (Supplementary Figs 5 and 6). The mean was almost 0.125, as expected (Fig. 5a). However, we determined that the HO ratio was slightly higher, while that of TC was lower, possibly because of a distortion of genetic kinships among the eight founders. IR8 and IR24 are common founders of TK, SU, MI and HO (Supplementary Fig. 7). SU is a founder of both MI and HO. BE and TC have a common founder, Ouu 342. Therefore, the haplotypes might not be predicted accurately in regions of the same origin. However, this inaccuracy would not affect the genetic studies because the same origin provides the same allele.

**Figure 6:**
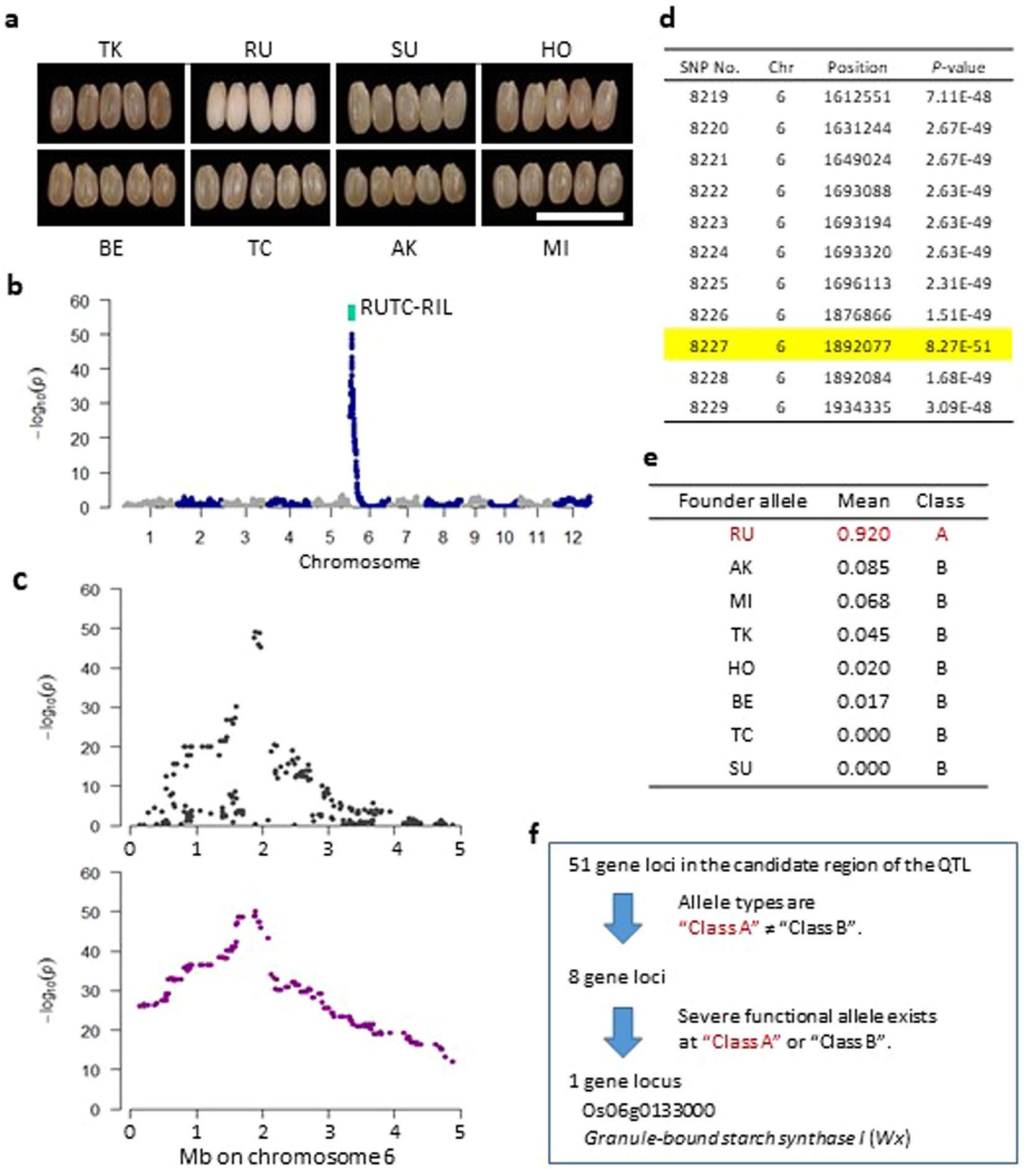
Haplotype-based allele mining of a gene for glutinous endosperm. (**a**) Representative hulled grains of the eight founders. (**b**) Manhattan plot of the genome-wide association study (GWAS) of glutinous endosperm using haplotype data. A region including a GWAS peak on chromosome 6 was also detected by QTL analysis in RUTC-RILs. (**c**) Pattern differences between SNP-(top) and haplotype-based GWAS (bottom). Manhattan plot views of the GWAS on chromosome 6 (0 to 5 Mb). (**d**) Candidate region of a gene for glutinous endosperm. The SNP with the lowest *P*-value (No. 8227) is highlighted in yellow. (**e**) Classification of founder alleles on the basis of pairwise Mann–Whitney *U-*tests with controlled family-wise error rate (*P* < 0.01). (**f**) Narrowing down the candidate genes in the QTL region.

To examine the genotypes of the JAM lines from another perspective, we investigated the proportions of haplotypes in each JAM line (Fig. 5b). Although the haplotype proportions from *japonica* (AK, BE, MI and TC) and *indica* (HO, RU, SU and TK) ranged from 0.15 to 0.96 and from 0.03 to 0.84, respectively, the median values in both *japonica* and *indica* founders were approximately 0.5, and the proportion of *japonica* or *indica* founders ranged from 0.3 to 0.7 in 350 (93%) of the 376 JAM lines. Next, we checked the rate of one qualitative trait, glutinous endosperm, derived from RU (see Fig. 6a). The glutinous endosperm trait appeared in 57 (18.9%) of the 358 JAM lines. The chi-square test (*P*-value = 0.0503) supported the null hypothesis, meaning that the appearance of the trait was not due to distorted segregation at the locus for glutinous endosperm. These results confirmed that the JAM population has almost equal proportions of the backgrounds of the founders.

### Allele information of annotated genes in the eight founders

We analysed short reads to obtain allele information for all genes. We categorized the effects of mutations on gene function into four classes using SnpEff software^20^ and categorized genes into allele types by considering the effects and the number of variations. A *japonica* Nipponbare allele and similar alleles with fewer than two variations were always placed into Cluster 1. The number of clusters can be as high as 8 because of the presence of eight founders, and the presence of a given allele in a higher number of clusters indicates a greater difference from the Nipponbare allele. The distribution of gene allele types differed among the founders (Fig. 2b, Supplementary Dataset 1), implying the potential presence of various alleles in the JAM population. A large proportion of genes in the four *japonica* cultivars were classified into Cluster 1, and more than half of the genes of the four *indica* cultivars were classified into Clusters 3–5 (Fig. 2b). These results revealed the positions of *japonica* and *indica* gene alleles and their blocks (Fig. 2c). For example, Cluster 1 genes of SU, TK and HO appeared to be in the centromeric regions of chromosomes 5 and 8, explaining the relatively low SNP density detected by GBS analysis in these regions (Fig. 4a).

### Haplotype-based allele mining for glutinous endosperm and culm length

To determine whether the detection power of GWAS was higher with haplotype data than with data on 16,345 SNPs, we chose glutinous endosperm as a qualitative trait and CL as a quantitative trait.

**Figure 7:**
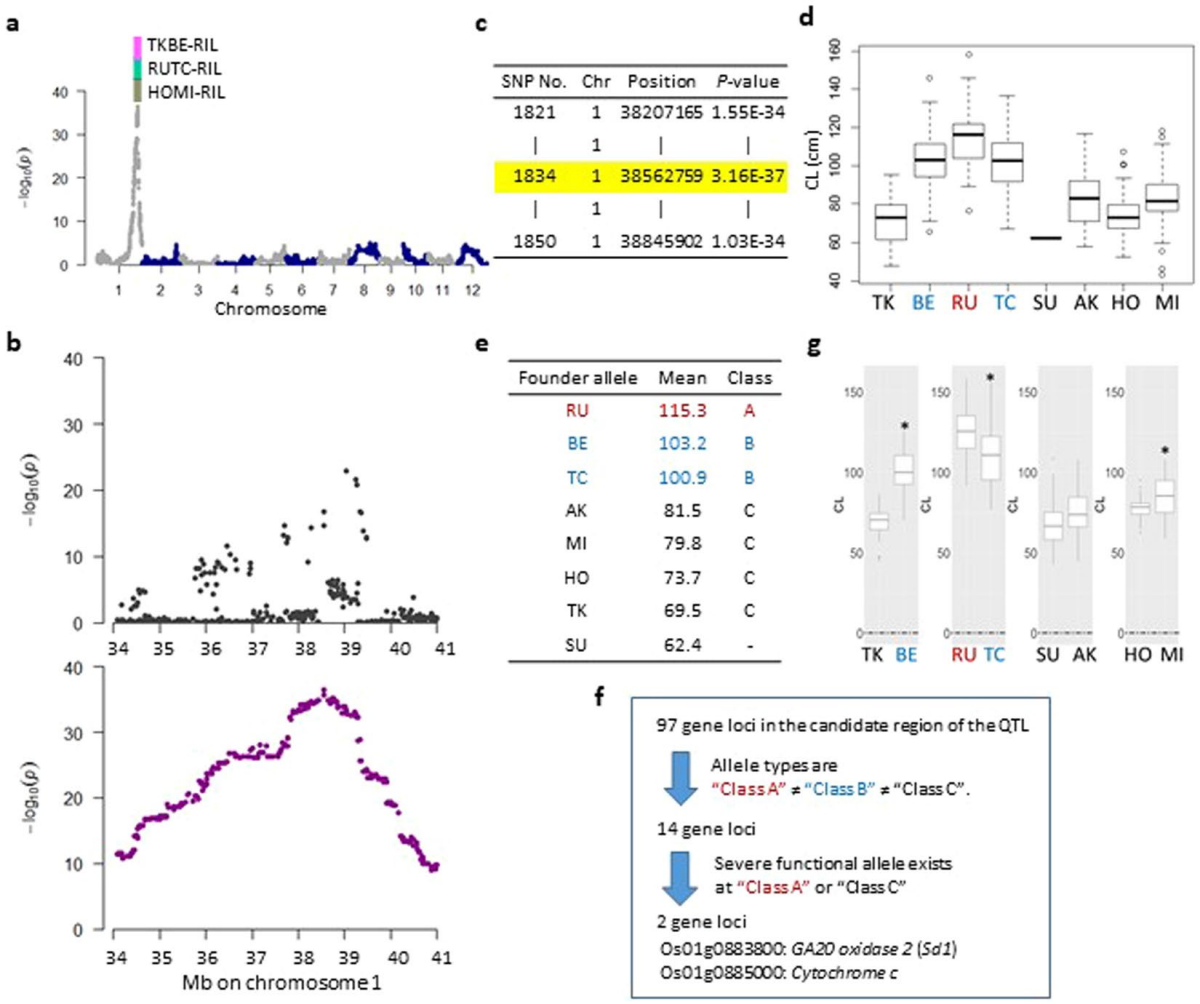
Haplotype-based allele mining of gene(s) regulating CL. (**a**) Manhattan plots of GWAS for CL using haplotype data. A region including a GWAS peak on chromosome 1 was also detected by QTL analysis in TKBE-, RUTC- and HOMI-RILs. (**b**) Pattern differences between SNP- (top) and haplotype-based GWAS (bottom). Manhattan plot views of GWAS on chromosome 1 (34 to 41 Mb). (**c**) Candidate region (SNP Nos. 1821–1850) of a QTL regulating CL. The SNP with the lowest *P*-value (SNP No. 1834) is highlighted in yellow. (**d**) Box plots of CL at SNP No. 1834 for the allele of each founder. (**e**) Categorization of founder alleles into three classes on the basis of pairwise Mann–Whitney *U*-tests with controlled family-wise error rate (*P* < 0.01). (**f**) Narrowing down the candidate genes in the QTL region. (**g**) Box plots of CL at the QTL region in the RILs. Asterisks indicate significant differences between the two founders (*t*-test: *P* < 0.01).

Among founders, only RU had glutinous endosperm, leading to white dehulled rice (Fig. 6a). We denoted glutinous as 1 and non-glutinous as 0 and applied GWAS to the glutinous endosperm trait using the SNP and haplotype data of the JAM population. The results revealed a major peak on the short arm of chromosome 6 (Fig. 6b). In the Manhattan plot, the plots were smoother with the haplotype data than with the SNP data (Fig. 6c). The GWAS of CL revealed a major peak on chromosome 1 (Fig. 7a). The Manhattan plot using haplotype data was more continuous than the plot using SNP data (Fig. 7b). The patterns of these plots were more in line with the haplotype data than with the SNP data because the *P*-value also decreased, showing a stronger peak when distance from the causal gene decreased. Moreover, quantile-quantile (Q-Q) plots in the GWAS using the haplotype data for glutinous endosperm and CL showed more continuous, compared to the SNP data, and the deflection from each null line started earlier (Supplementary Fig. 8). That might not be attributed to background noise but the wider range of SNPs where *P*-values are continuously low at the peaks of the Manhattan plot (Figs 6c and 7b). These findings support the suitability of the haplotype data for GWAS.

Next, we checked whether gene allele type information was useful for searching genes. In GWAS for glutinous endosperm, the lowest *P*-value was detected at SNP No. 8227 (Fig. 6d). We defined the loci for glutinous endosperm (SNP Nos 8219–8229) as a region with *P*-values lower than the lowest *P*-value multiplied by 10^3^. The haplotype of RU and those of other founders at the SNP position with the lowest *P-*value were categorized into different phenotypic classes on the basis of pairwise Mann–Whitney *U*-tests with a controlled family-wise error rate (*P* < 0.01, Fig. 6e). For 8 of the 51 genes in the loci, the gene allele types differed between RU (Class A) and the other cultivars (Class B) (Fig. 6f). To further narrow down the candidate genes for glutinous endosperm, we searched genes that included the severe gene allelic variation categorized into “variants_impact_HIGH” using SnpEff software^20^. The only gene with severe allelic variation in Class A or B was *Wx*, a gene coding for glutinous endosperm^21^. In the *Wx* locus, only RU had an in-frame 23-bp insertion in the 2nd exon, which created a premature stop codon^22–24^ (Supplementary Fig. 9a). QTL analysis using RUTC-RILs validated the *Wx* locus (Fig. 6b, Supplementary Table 1).

In GWAS for CL, the lowest *P*-value was detected at SNP No. 1834 (Fig. 7c). We defined the QTL region (SNP Nos. 1821–1850) using the criterion above. At SNP No. 1834, three classes were categorized: RU (Class A), BE and TC (Class B), and the other founders (Class C) (*P* < 0.01, Fig. 7d,e). Unfortunately, SU could not be classified because only one JAM line had the SU haplotype at this position. Of the 97 loci in the QTL region for CL, 14 differed among the allele types (Fig. 7f). Two genes, *Sd1* and *Cytochrome c*, were selected in a search for the allelic variation that can affect the gene function in Class A (RU) or Class C (AK). *Sd1* controls gibberellin biosynthesis^25^, although there is no information on the role of *Cytochrome c* in CL. Sequence data revealed four types of *Sd1* alleles in the three classes: Class A was *indica* type (amino acid substitution Q273R), Class B was *japonica* Nipponbare type, and Class C included the IR8-type allele (AK, SU, HO and TK: more than 310-bp deletion from exon 1 to exon 2 and Q273R) and the Reimei allele (MU: D282H)^26,27^ (Supplementary Fig. 9b). The IR8 allele is considered to be null, and the IR8 and Reimei alleles are used to develop *indica* and *japonica* cultivars with a semi-dwarf phenotype^26^. QTL analysis for CL in the four types of RILs detected QTLs in the region that includes the two genes (Fig. 7a, Supplementary Table 1). The patterns of CL in the four RILs according to founders supported the three classes (Fig. 7g). These results indicate that the gene allele type information of the eight founders is effective for narrowing down the candidate genes in the loci.

## Discussion

In this study, we produced a JAM population derived from eight high-yielding Japanese cultivars and developed a novel method for allele mining. This method is based on the haplotype blocks predicted using 16,345 SNPs data and gene allele information of the founders provided from NGS data (Supplementary Fig. 10).

We identified 16,345 SNP alleles through GBS and subsequent *in silico* analyses and selected SNPs according to strict criteria (minor allele frequency of more than 0.0625 and more than 95% actual data). Previously, to distinguish the genomes of the eight founders, we used 4,653 markers^28^; here, we obtained three times as many markers. In an *indica* 8-way MAGIC population^9^, GBS analysis provided 17,387 SNPs, but the criteria (minor allele frequency of more than 0.05 and more than 60% of actual data) were less strict. In a subsequent report^10^, an Illumina Infinium rice 6 K SNP chip was used to genotype the same population. Given the imputation inaccuracy in haplotype estimation, SNPs with high amounts of missing data should be eliminated. Therefore, it was appropriate to apply strict criteria to the selection of SNPs to ensure the reliability of GWAS with the JAM population. The SNPs were used for haplotype prediction of JAM lines. The rate of glutinous endosperm in the JAM lines with the SNP with the lowest *P*-value in GWAS was close to 1 (RU = 0.920, Fig. 6), indicating the high accuracy of haplotype prediction.

The pattern of LD decay in JAM over the whole genome revealed no large LD blocks other than the centromeric region of each chromosome; LD sizes varied among chromosomes. Japanese high-yielding rice cultivars have common origins and biased genome segments because of phenotypic selection for yield-related traits^29^. Further, different recombination rates among chromosomes might influence the LD patterns.

For the qualitative and quantitative traits, GWAS based on the haplotype data showed smoother *P*-value plots, in contrast to ordinary GWAS based on 16,345 SNPs. One possible explanation for this difference is as follows. The allelic effects of RU and the other three *indica* (HO, TK and SU) SNPs are different in the loci for the glutinous endosperm and CL phenotypes. In the loci and the surrounding region, both *indica* cultivar-shared SNPs and RU-specific SNPs were aligned. In the cases of glutinous endosperm and CL, the allelic effects of *indica* SNPs were cancelled due to different phenotypes, indicating no contribution of *indica* SNPs for the detection of the loci for the phenotypes. However, with an analysis based on haplotypes instead of SNPs, all SNP information has a potential contribution. We suggest that the conversion of SNP data to haplotype data is a key step to accurately define the QTL position in GWAS.

We showed that the allele information for all annotated rice genes was useful for narrowing down the causative genes of glutinous endosperm and CL. Gene-based association analysis of 176 *japonica* rice varieties reportedly worked well to identify candidate genes for the traits of interest^30^, indicating that such analysis is effective for mapping candidate genes. Genome resequencing of *japonica* rice varieties enabled gene-based association analysis, the best method using currently available techniques. However, in the present study, we used resequencing data for only the eight founders and performed GBS analysis of the JAM population. This approach was suitable and cost effective because the genomes of the JAM lines are derived from those of the founders and resequencing all of the JAM lines would be redundant.

In GWAS for CL, we detected one of the candidate genes, *Sd1*, and three classes of *Sd1* alleles. These results indicate that the JAM population is useful for revealing the effects of multiple gene alleles. The allelic effect of a QTL is usually determined by evaluating near-isogenic lines, but establishing these lines requires several backcrosses. Phenotypic and genotypic analyses of MAGIC populations provide an alternative method to investigate gene allele functions. We confirmed two advantages of MAGIC lines—greater phenotypic diversity and a higher number of alleles—in comparison with RILs. The high number of alleles is particularly important for gene detection in genetic studies. Our QTL analysis using bi-parental RILs detected no QTL for CL in SUAK-RILs and detected the QTL for glutinous endosperm only in RUTC-RILs. These results emphasize that QTL analysis can miss QTLs in bi-parental RILs in which allelic functions of causal genes are similar or identical. In fact, the *Wx* alleles of all founders (except RU) and the *Sd1* alleles of SU and AK belong to the same allele types. These results indicate that the genetic approach using MAGIC populations identifies more allele types and reduces the omission of highly effective genes.

The concept of allele mining in the JAM population using the haplotype-based allele mining (HAM) method would be applicable to the other MAGIC populations and nested association mapping populations. Our next target in research on the JAM population is to identify the alleles involved in grain yield. We will examine whether the HAM method is useful for elucidating this complex trait in the JAM population. Searching for genes regulating complex traits such as biomass and grain yield remains problematic because of the involvement of many weak alleles, the identification of which is hindered by high LD in local contexts. To further decrease LD in the JAM population, we are producing a concurrent crossed population for use in additional trials.

## Methods

### Artificial pollination of rice

To emasculate unpollinated spikelets, they were kept in a water bath at 43 °C for 7 min; the top third or half of the hull was then cut by scissors, and all anthers were removed with a pipette tip under vacuum. The pollen of vibrant yellow dehiscent anthers was used to pollinate the emasculated spikelets directly or with forceps. To prevent further pollination, panicles with pollinated spikelets were covered with envelopes for approximately 5 days, and mature seeds were collected 30 to 40 days later.

### Cultivation of JAM lines and RILs and trait assessment

Germinated seeds were sown in trays filled with soil on 9 May 2016 and incubated at 30 °C in the dark for 2 days. Seedlings were grown in a Tsukuba Kannondai paddy field covered with thin plastic film to protect the plants from the cold. Individual seedlings were transferred to another paddy field on 8 June 2016 and cultivated according to standard procedures in NARO in Tsukuba, Japan. DTH was scored as the number of days from sowing to the appearance of the first panicle. CL was assessed as the length of the longest culm in each plant, which was measured with a ruler more than 10 days after heading. DTH values were binned into 5 categories and CL values into 6 categories; these categories were then used to generate heat maps. Glutinous endosperm was evaluated based on the colour of dehulled rice. We used 376 JAM lines, which were selected with consideration of kinship. As explained above, we attempted to produce 1,000 JAM lines by developing 10 lines from each eight-way recombinant. We picked up 3 to 4 lines (F5) derived from each eight-way recombinant to select the 376 JAM lines. We used 144 HOMI-RILs, 138 TKBE-RILs, 81 RUTC-RILs and 132 SUAK-RILs.

### NGS DNA sequencing of the eight founders, classification of gene allele types and hierarchical clustering

We performed short-read Illumina resequencing for AK, BE and RU and deposited the data in the DDBJ Sequence Read Archive under the accession number DRA005784. For the other five founders, we used the resequencing data we reported previously^29^. Low-quality bases and the adapters in each read were trimmed using Trimmomatic software^31^. Trimmed reads were mapped to the Nipponbare International Rice Genome Sequencing Project (IRGSP) v.1 reference genome using BWA software with default settings^32^. Only uniquely mapped reads with a mapping quality score of ≥20 were sorted and indexed using SAMtools software^33^. To improve the raw alignments around insertion and deletion mutations (indels), local re-alignments were performed using GATK software^34^ (https://software.broadinstitute.org/gatk/). PCR duplicates were removed using Picard software (http://picard.sourceforge.net). Indels and SNPs were identified individually for each sample using both SAMtools and GATK software. Homozygous SNPs found in the eight founders were used as reference SNPs to predict haplotypes in the JAM population.

Non-homozygous and low-depth (<3-fold) variants were removed, and the remaining variants were used to analyse their effects on gene function in SnpEff software^20^. This software provides the number of variations that belong to one of the four categories (variants_impact_high, _moderate, _low or _modifier) according to their putative effect. We classified all annotated gene loci (42,795 loci) into allele types according to the number of alleles in each SnpEff category. Nipponbare alleles and similar alleles were in Cluster 1. The maximum number of clusters was eight because of the eight founders; a higher cluster number indicated a greater difference from the Nipponbare allele. The data on the clusters of all annotated genes in the eight founders are summarized in Supplementary Dataset 1. Hierarchical cluster classification of the founders based on the gene clusters was performed using the R function “hclust” with Ward’s method^35^.

### GBS, LD analysis and haplotype prediction of JAM lines

Total DNA was extracted from leaf blades of the 376 JAM lines and 8 founders. The genotyping-by-sequencing (GBS) of the 376 JAM lines and founders was undertaken at Beijing Genome Institute (BGI, Shenzhen, China) using previously described methods^36^. The outline of GBS procedure is described as follows. After check of sample integrity and purification detected by agarose gel electrophoresis, GBS library was constructed. 100 ng DNA was digested by restriction enzyme, ApeKI (NEB, Ipswich, Massachusetts, USA), then added common adapter and barcode adapter in ligation reaction mix. Ligate products were pooled and purified with QIAquick PCR Purification Kit (Qiagen, Valencia, California, USA). Several rounds of PCR amplification with PCR Primer Cocktail and PCR Master Mix were performed to enrich the Adapter-ligated DNA fragments. Then, the PCR products were selected by running another 2% agarose gel to recover the target fragments. Sample gels were purified with QIAquick Gel Extraction kit (Qiagen, Valencia, California, USA). The final library was quantitated in two ways: Determine the average molecule length using the Agilent 2100 bioanalyzer instrument (Agilent Technologies, Santa Clara, California, USA) and quantify the library by real-time quantitative PCR (TaqMan Probe, Applied Biosystems, Foster City, California, USA). The qualified libraries were amplified to generate the cluster on the flowcell and the amplified flowcell was sequenced pair end on the Illumina HiSeq. 4000 System. To obtain the clean GBS data, preprocessing was performed as next four steps. 1) Remove reads with adaptors; 2) Remove low quality reads (i.e. reads with more than 50% bases whose quality value are less than or equal to 10); 3) Remove reads in which unknown bases are more than 10%; 4) Remove reads which do not contain barcode, trim the barcode, and then remove reads lacking key sequence of the enzymes.

The clean GBS data (an average of 667 Mb of 100-bp short reads per sample) obtained were analysed in a similar manner to the NGS analysis of the founders’ genomes. We extracted only high-quality homozygous SNPs with two or more reads (DP, ≥2-fold; FQ, ≥25; 16,345 SNP alleles in total). Heterozygous genotypes were considered null. The imputation produced the SNP set for LD and haplotype analysis of the JAM population.

LD heatmaps and LD decay plots were produced in the R package popgen (http://www.stats.ox.ac.uk/~marchini/software.html). A total of 2,355 randomly selected alleles (1/7 of all SNP alleles) were mapped on the physical map due to the reduction in calculation time. Physical positions are indicated as the cumulative positions (Mb) from chromosome 1 to 12.

We estimated the parental haplotypes in the JAM lines using the method proposed for haplotype prediction in an Arabidopsis MAGIC population^2^ (http://mtweb.cs.ucl.ac.uk/mus/www/19genomes/magic.html). All parameters were set to their defaults, except “-p”, which was set to 2. Proportions of haplotypes from the eight founders were calculated in the JAM population and each JAM line.

### Development of indel markers and genotyping of RILs

To determine the parental origins of variations in RILs, indel markers were designed based on large indel regions (≥10 bp) with high sequencing depth (DP, ≥5-fold). Primer pairs for the selected indel regions were automatically designed using a Perl script to control the Primer3 core program^37^. PCR products ranged from 80 to 150 bp.

TKBE-, SUAK- and HOMI-RILs were analysed using 177, 176 and 167 indel markers, respectively. PCR with a KAPA2G Fast PCR kit (Roche, Basel, Switzerland) and DNA electrophoresis in 3% agarose gel were performed as previously described^38^. The SNPs of RUTC-RILs were checked using the Golden Gate detection system (Illumina, San Diego, California, U.S.) with the Fcore SNP set^39^. We checked all SNPs with GenomeStudio (Illumina) and chose 437 SNPs for the QTL analysis of DTH and CL; the SNP selection criteria have been described previously^40^.

### GWAS and QTL analysis

GWAS was performed using R software. Associations of the imputed genotype data with phenotype data for glutinous endosperm and CL were analysed using the population structure (Q)+ Kinship (K) model^41^ to calculate the *P*-values using the “rrBLUP” package. Spots of *P*-values were illustrated in Manhattan plots using the “qqman” package. *Q*-values were calculated from the *P*-values corrected with Bonferroni’s adjustment and were used to produce Q-Q plots.

For QTL analysis for glutinous endosperm and CL in the four types of RILs, we constructed genetic maps using version 3.0b of MAPMAKER/EXP software^42^. The QTL analysis was performed by interval composite mapping using version 2.5 of QTL Cartographer software, and the threshold was calculated from 1,000 permutations^43^.

## Electronic supplementary material

Supplementary Information

Dataset 1
