# Supplementary material for: Haplotype-based allele mining in the Japan-MAGIC rice population
Source: Sci Rep. 2018 Mar 12;8:4379. doi: 10.1038/s41598-018-22657-3 (PMC5847589; doi:10.1038/s41598-018-22657-3)
Supplement: Supplementary file 1 — Supplementary Information [file 41598_2018_22657_MOESM1_ESM.pdf]

Haplotype-based allele mining in the Japan-MAGIC rice population

Daisuke Ogawa[1,2], Eiji Yamamoto[2], Toshikazu Ohtani[2], Noriko Kanno[1,2], Hiroshi Tsunematsu[1], Yasunori Nonoue[1], Masahiro Yano[1,2], Toshio Yamamoto*[1,2], Jun-ichi Yonemaru*[1,2]

Affiliations:

[1] Institute of Crop Science, National Agricultural and Food Research Organization (NARO), Japan

[2] Agrogenomics Research Centre, National Institute of Agrobiological Sciences (NIAS), Japan

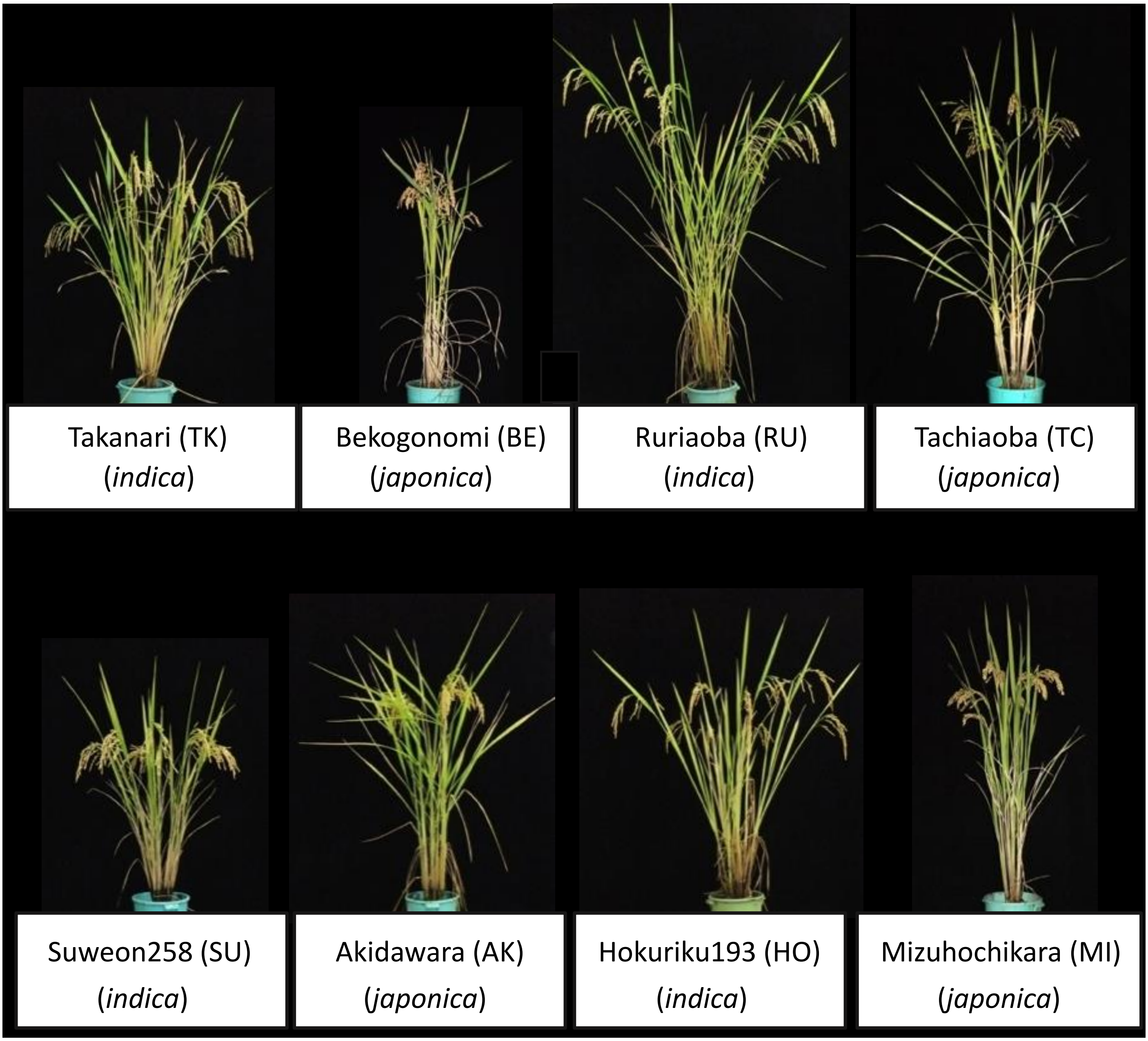

Supplementary figure 1. The eight founders of the JAM population.

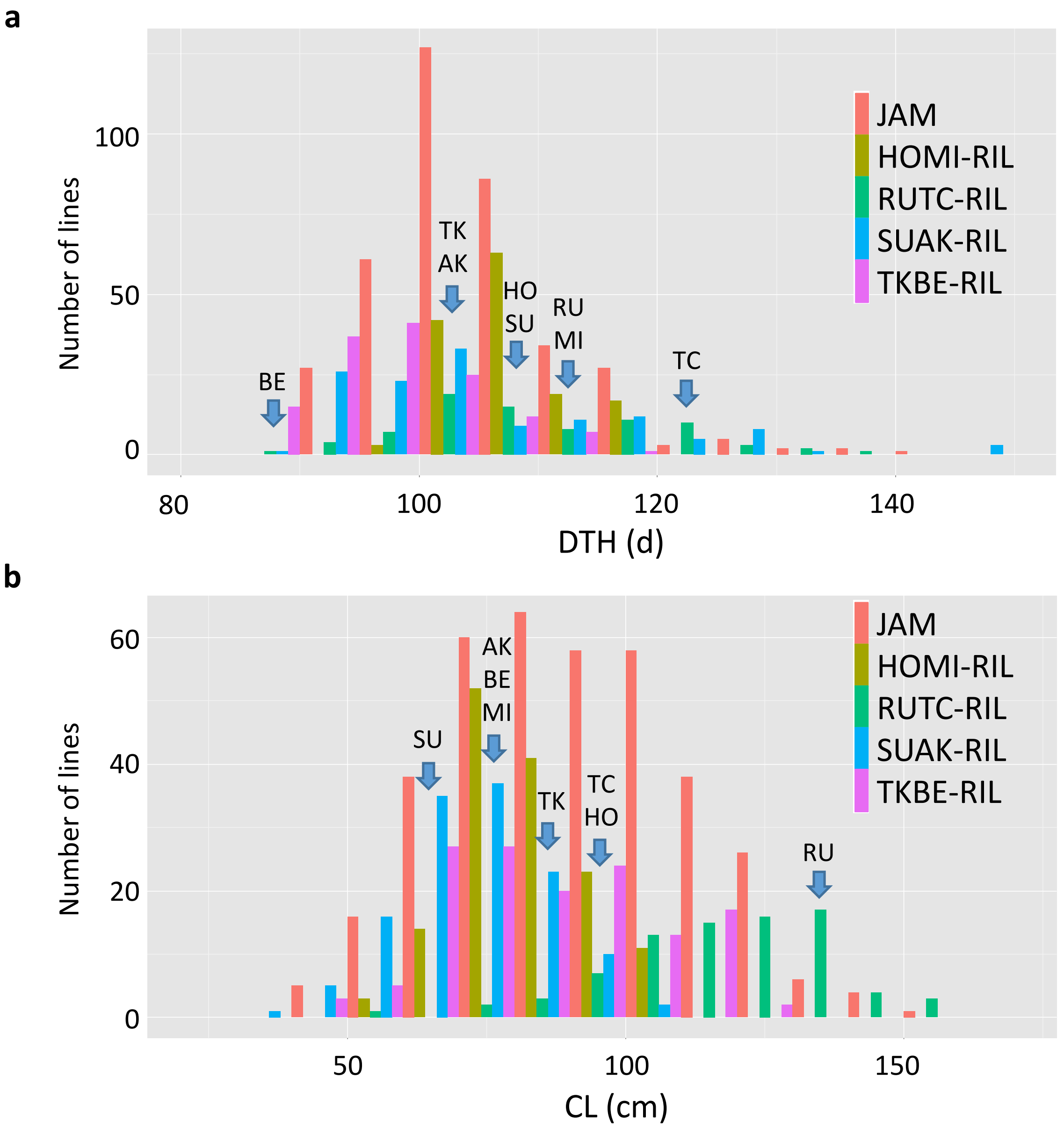

Supplementary Figure 2. Phenotype distribution in 376 JAM lines and 4 RIL types. (a) Days to heading (DTH) from sowing. (b) Culm length (CL) measured more than 10 days after heading. Arrows indicate the values of the eight founders.

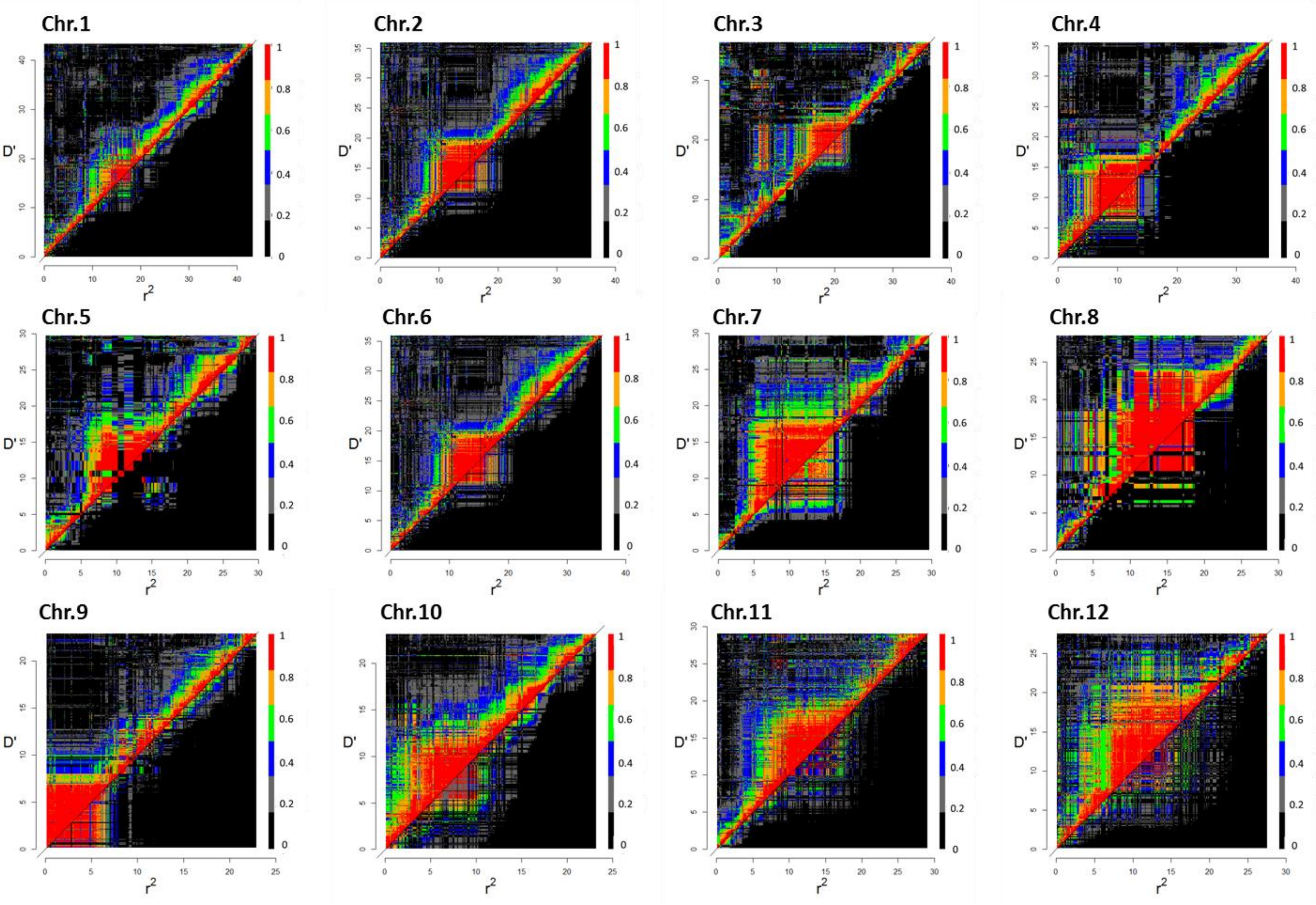

Supplementary Figure 3. Heat maps of linkage disequilibrium in each chromosome of the 376 JAM lines. Physical position is indicated in Mb. D’ and r^2 are shown above and below the diagonals, respectively.

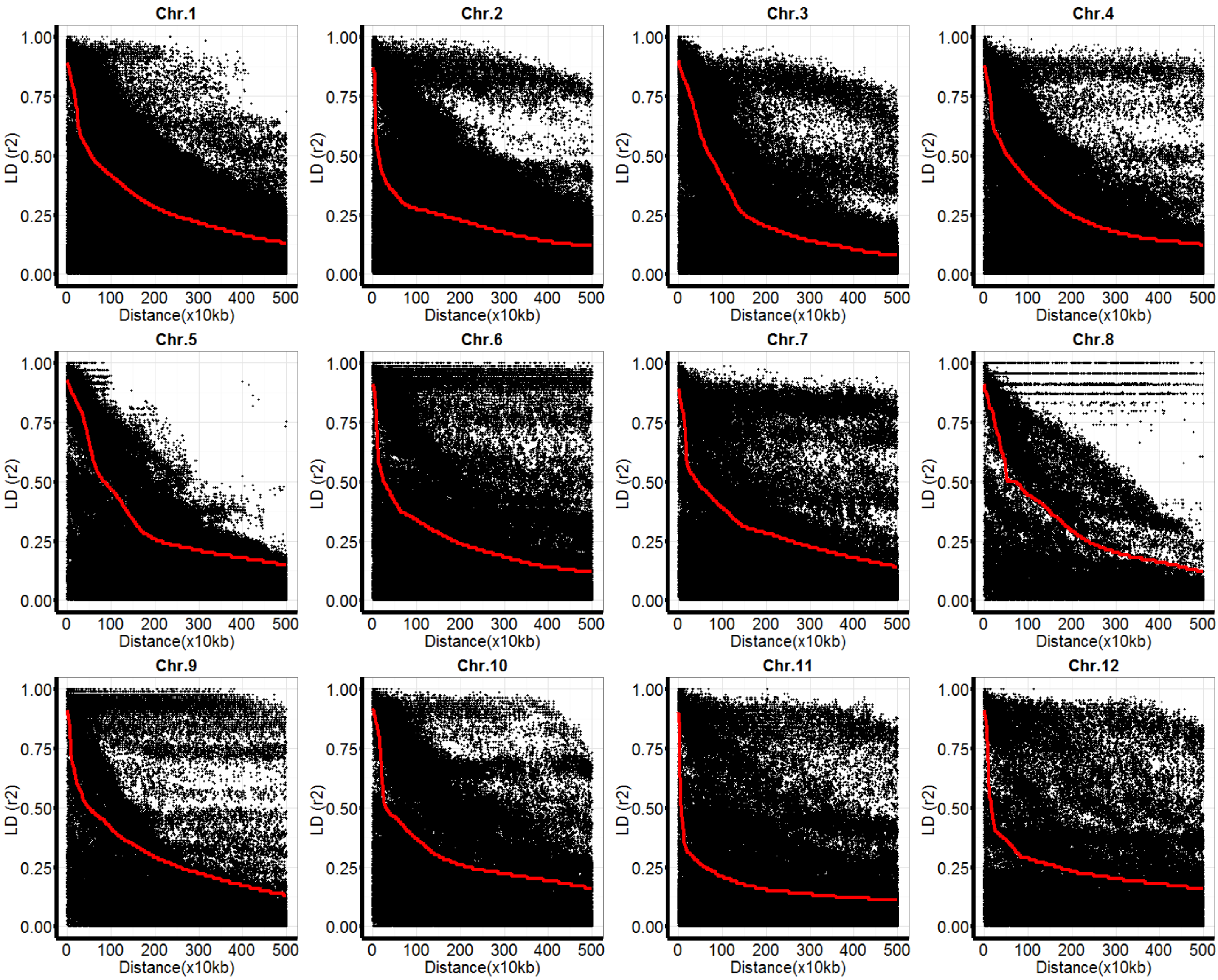

Supplementary Figure 4. Linkage disequilibrium (LD) between SNP pairs within a 5-Mb window on each chromosome of the 376 JAM lines. Red lines show median values in 10-kb windows.

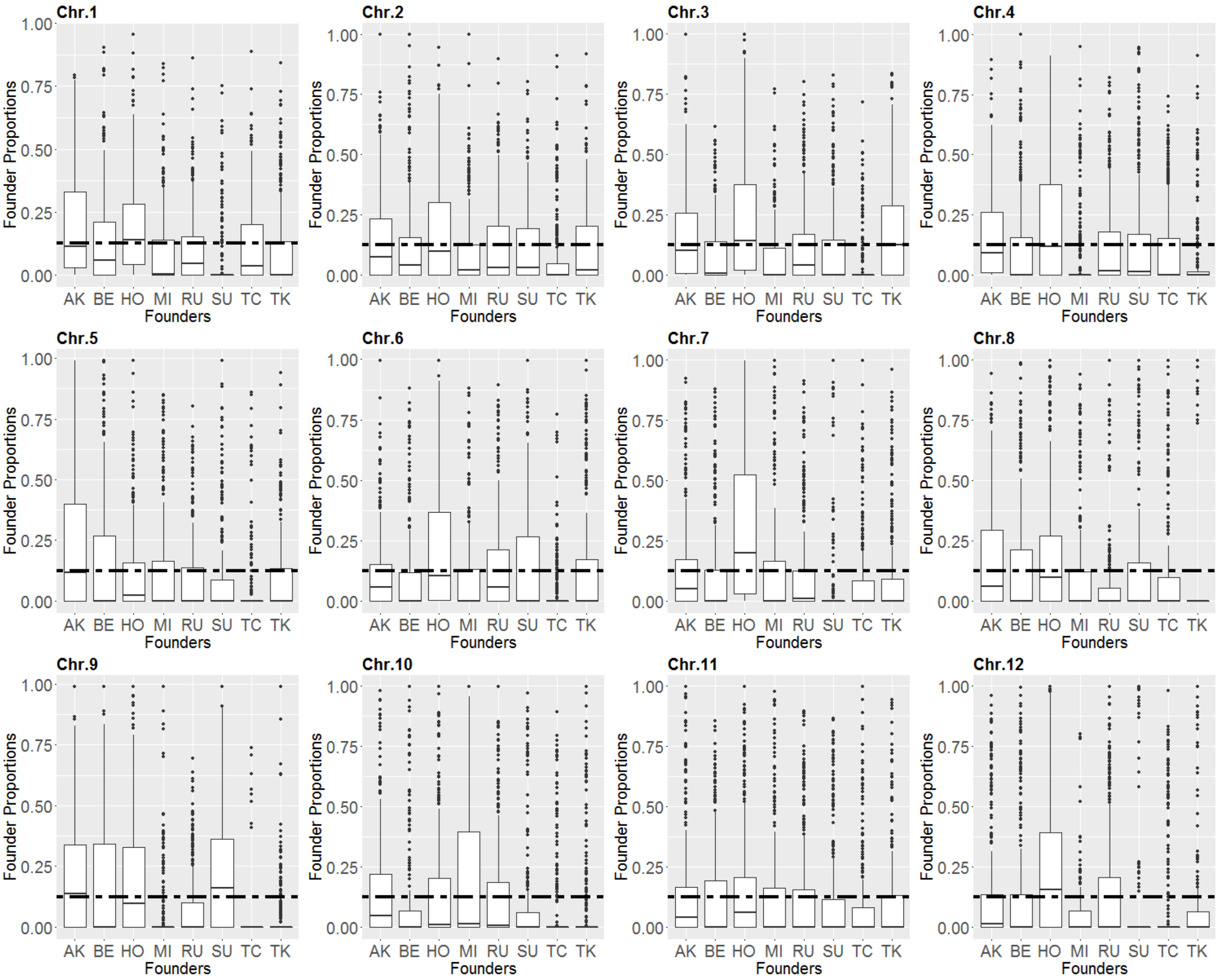

Supplementary Figure 5. Box plots of the founder genomes in each chromosome of the 376 JAM lines.

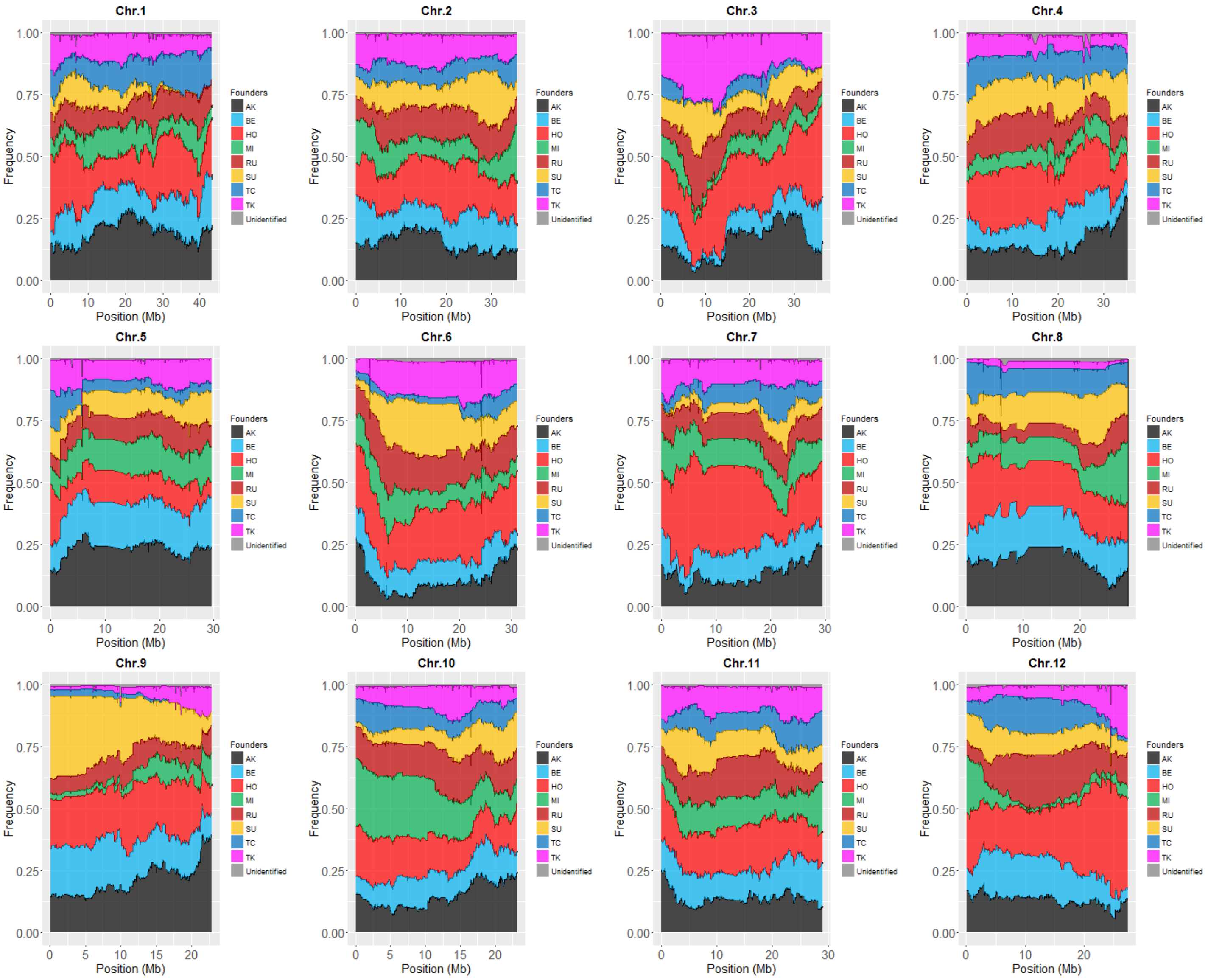

Supplementary Figure 6. Distribution of haplotypes on each chromosome of the 376 JAM lines.

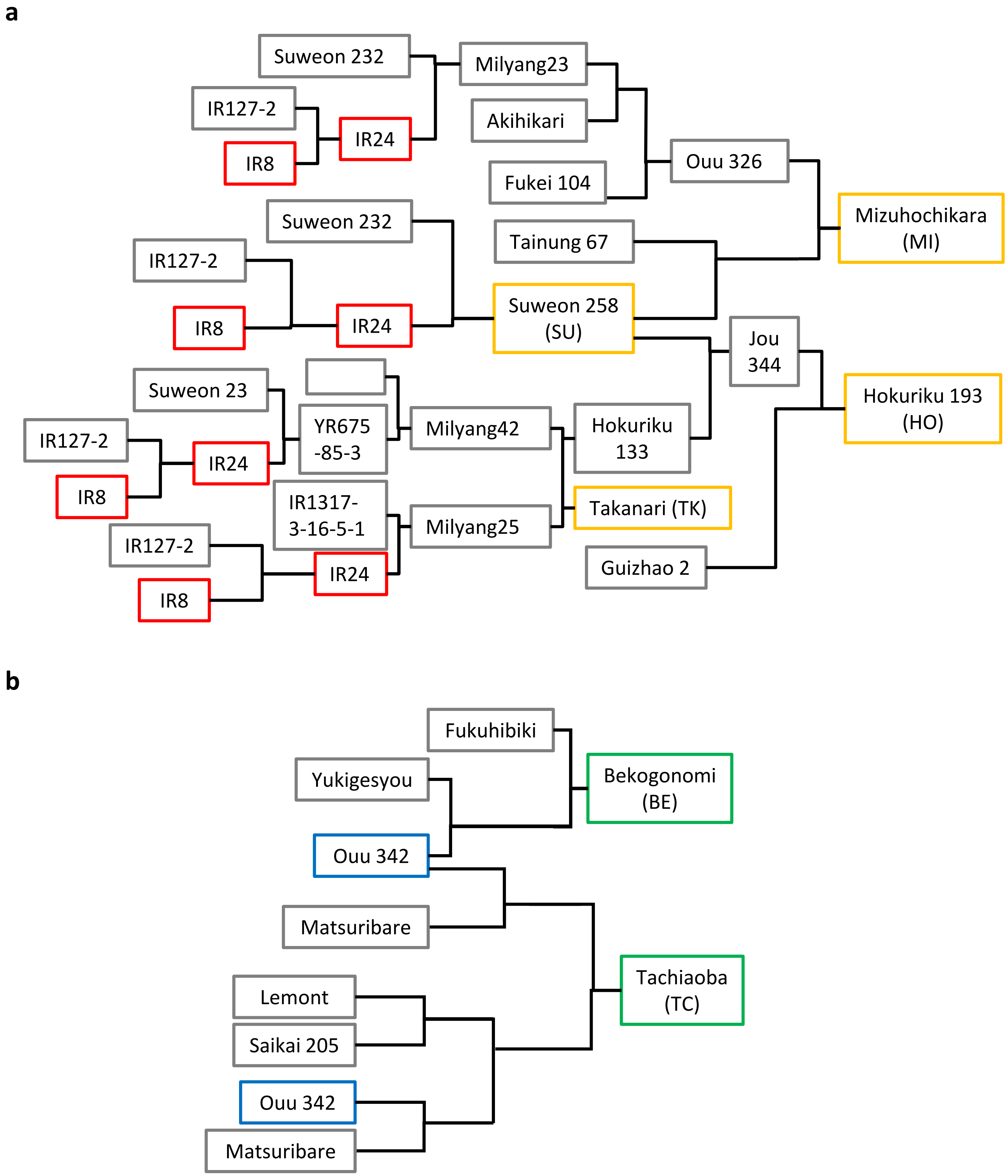

Supplementary Figure 7. Pedigrees of some of the founders. (a) IR8 is a common ancestor (via IR24) of MI, HO, SU and TK. (b) Ouu 342 is a common ancestor of BE and TC. Blank indicates no information.

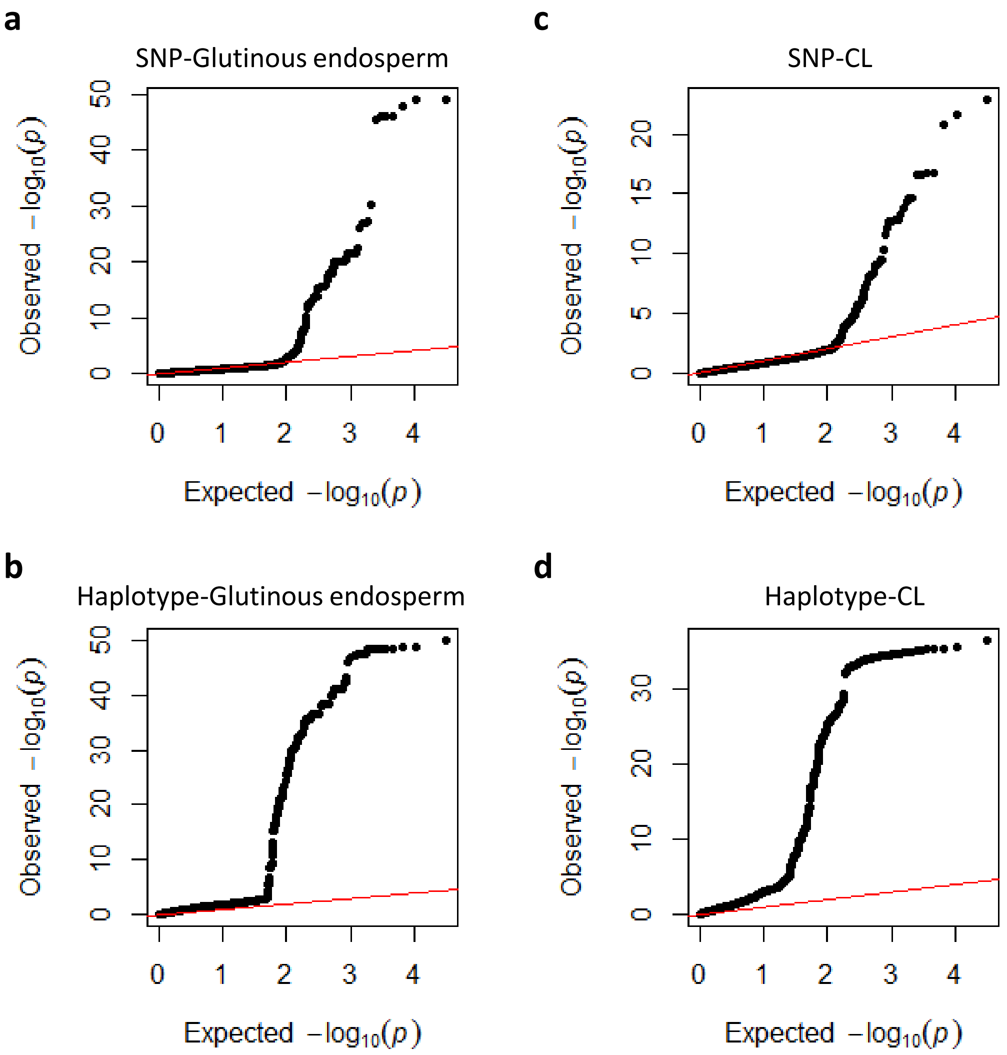

Supplementary Figure 8. Q-Q plots of genome-wide association for (a, b) glutinous endosperm and (c, d) CL. GWAS was performed with (a, c) 16,345 SNPs and (b, d) haplotype data. The red line shows the ideal normal distribution.

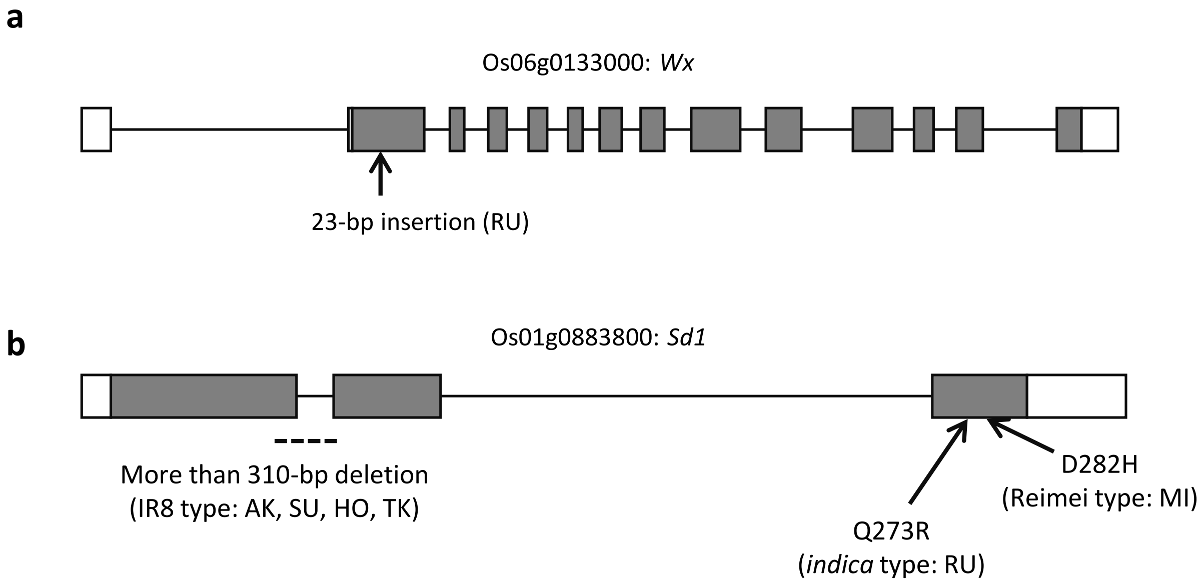

Supplementary Figure 9. Variations of the *Wx* and *Sd1* loci in the eight founders. Grey boxes, exons; black bars, introns; white boxes, untranslated regions. (a) *Wx* gene structure. Arrow shows the position of a 23-bp insertion (non-functional *wx*) in RU. (b) *Sd1* gene structure. Three variations (IR8, *indica* and Reimei types) are shown.

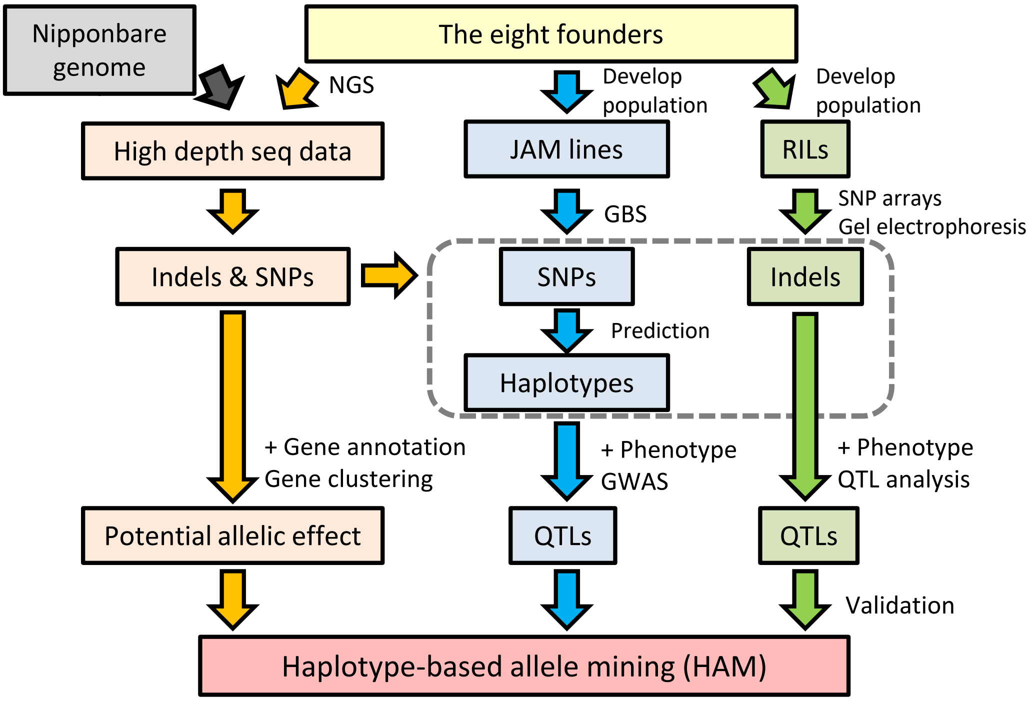

Supplementary Figure 10. Haplotype-based allele mining (HAM) in the JAM population. Orange path: next-generation DNA sequencing (NGS) data of the genomes of the eight founders were used to find SNPs and insertion and deletion mutations (indels) to define allele types and to estimate the effect of each potential allele. Blue path: genotyping-by sequencing (GBS) analysis reveals SNP data of JAM lines. The SNP data were summarized into haplotype data. GWAS was performed using the haplotype and phenotype data of the JAM population. Genes in the identified QTL regions were searched for their potential allelic effects. Green path: QTL data from the genetic study in RILs were used to validate QTLs detected in the JAM population. Dashed contour: SNP data and indels obtained from NGS analysis were used for the genotypic analyses in the JAM lines and RILs.

Supplementary Table 1. Putative QTLs for glutinous endosperm and culm length detected in the four types of RILs.

Traits	RILs	Chr	Closest marker position (Mb)[a]	LOD	Additive effect[b]	$R^{2\ c}$
Glutinous endosperm	RUTC	6	0.80*	9.7	-0.4	0.478
Culm length	HOMI	1	36.76	6.9	4.8	0.166
Culm length	HOMI	5	22.596	3.3	-3.1	0.075
Culm length	HOMI	6	28.046	7.2	-4.5	0.161
Culm length	HOMI	9	16.42	3.0	2.9	0.058
Culm length	RUTC	1	40.25	9.3	-12.9	0.349
Culm length	RUTC	8	25.99	3.7	8.0	0.155
Culm length	SUAK	2	16.98	4.5	4.2	0.099
Culm length	SUAK	3	34.73*	12.5	7.2	0.289
Culm length	SUAK	6	8.43	9.0	6.8	0.175
Culm length	SUAK	6	24.45	3.3	-3.8	0.067
Culm length	TKBE	1	36.76	33.3	14.6	0.618
Culm length	TKBE	2	26.24	3.4	3.8	0.038
Culm length	TKBE	3	30.53	3.7	-3.9	0.044

a Physical positions of the closest markers to LOD peaks.

b Additive effect of alleles of japonica cultivar in each population.

c Percentage of the phenotypic variance explained by each QTL.

* Only QTL is shown at highest LOD peak value found in two QTL.
